# Prostate MRI learning curves: establishing training benchmarks for radiology and urology trainees

**DOI:** 10.1007/s00330-025-12177-w

**Published:** 2025-12-16

**Authors:** Pavel Stegarescu, Egon Burian, Amelie Lutz, Nathan Perlis, Ulrich Grosse, Nemanja Avramovic, Stoyan Benev, Constantin Bolz, Pia Götz, Marc Koschler, Joana Kostova, Ana Macek, Abigail Martin Mens, Khashayar Namdar, Ioan Popa, Aileen Satari, Sydney Schmidt, Feri Töckelt, Roman Wiegele, Jan Klein, Thomas Herrmann, Gustav Andreisek, Dominik Deniffel

**Affiliations:** 1 Department of Diagnostic and Interventional Radiology, Cantonal Hospital of Frauenfeld and Münsterlingen, Spital Thurgau AG, Frauenfeld, Switzerland; 2 Institute of Diagnostic and Interventional Radiology, University Hospital Zurich, Zurich, Switzerland; 3 Faculty of Medicine, University of Zurich, Zurich, Switzerland; 4 Department of Radiology, Stanford University School of Medicine, Stanford, CA USA; 5 Division of Urology, Department of Surgical Oncology, Princess Margaret Cancer Centre, University Health Network, Toronto, ON Canada; 6 Department of Diagnostic and Interventional Radiology, Eberhard Karls University Tuebingen, University Hospital Tuebingen, Tübingen, Germany; 7 Department of Nuclear Medicine, RWTH Aachen University Hospital, Aachen, Germany; 8 Department of Urology, Cantonal Hospital of Frauenfeld and Münsterlingen, Spital Thurgau AG, Frauenfeld, Switzerland; 9 Institute of Medical Science, University of Toronto, Toronto, ON Canada; 10 Department of Urology, Cantonal Hospital of Winterthur, Winterthur, Switzerland; 11 Division of Urology, Department of Surgical Sciences, Stellenbosch University, Western Cape, South Africa; 12 Hannover Medical School, Hannover, Germany; 13 Faculty of Medicine, TUM School of Medicine and Health, Technical University of Munich, Munich, Germany

**Keywords:** Prostate cancer, Multiparametric magnetic resonance imaging, Learning curves, Radiology training, Urology training

## Abstract

**Objectives:**

Evidence-based training benchmarks for prostate multiparametric MRI (mpMRI) interpretation remain undefined amid growing educational demands. We compared learning curves between radiology and urology trainees and quantified the impact of prior radiological experience.

**Materials and methods:**

Fourteen trainees (10 radiology, median 2.7 years experience; 4 urology, no imaging experience), all naïve to prostate mpMRI, prospectively interpreted 200 cases using a feedback-based platform. Performance metrics included agreement with expert consensus reference for PI-RADSv2.1 (≥ 3), PI-QUALv2 image quality, extraprostatic extension (EPE) grading, and readout time. Learning curves were modeled using generalized estimating equations; segmented regression identified inflection points; bootstrapping generated 95% CIs.

**Results:**

Prior radiological experience showed no significant impact on PI-RADSv2.1 (OR per year 1.06 [95% CI: 0.96, 1.16]) or PI-QUALv2 (1.05 [0.99, 1.23]), with a minor effect on EPE grading (1.11 [1.03, 1.24]). Final PI-RADSv2.1 agreement with reference was similar (urology 80.9%, radiology 77.4%; OR 1.24 [0.55, 3.10]), with sensitivity/specificity 0.84/0.80 and 0.83/0.79, and Cohen’s κ values (0.64 and 0.61) matching inter-expert κ = 0.63. Learning plateaued after 69–75 cases. Urology trainees demonstrated higher baseline PI-QUALv2/EPE agreement (OR 2.01 [1.35, 3.02] and 1.90 [1.11, 2.93]), but radiology trainees achieved similar final performance (PI-QUALv2: 88.0% vs 89.9%, OR 0.82 [0.35, 1.72]; EPE: 84.6% vs 90.0%, OR 0.61 [0.31, 1.42]). Readout times decreased markedly in both groups (final difference 53.3 s [−9.4, 95.9]).

**Conclusion:**

Feedback-based training enabled similar prostate mpMRI interpretation performance across specialties, with most learning within 75 cases. Prior radiological experience had a limited impact. These empirical benchmarks inform certification standards and early-residency curricula in radiology and urology.

**Key Points:**

***Question*** *Evidence-based training benchmarks for prostate mpMRI interpretation competency in radiology and urology trainees remain undefined, despite growing educational needs and clinical demands.*

***Findings*** *Learning curves of 200 prostate mpMRI cases with feedback showed radiology and urology trainees plateauing after 69–75 cases with similar PI-RADSv2.1, PI-QUALv2, EPE grading performance.*

***Clinical relevance*** *Our findings establish an empirical benchmark (~75 cases) to guide prostate mpMRI certification standards and support the implementation of training curricula early in residency across specialties, regardless of prior radiological experience.*

**Graphical Abstract:**

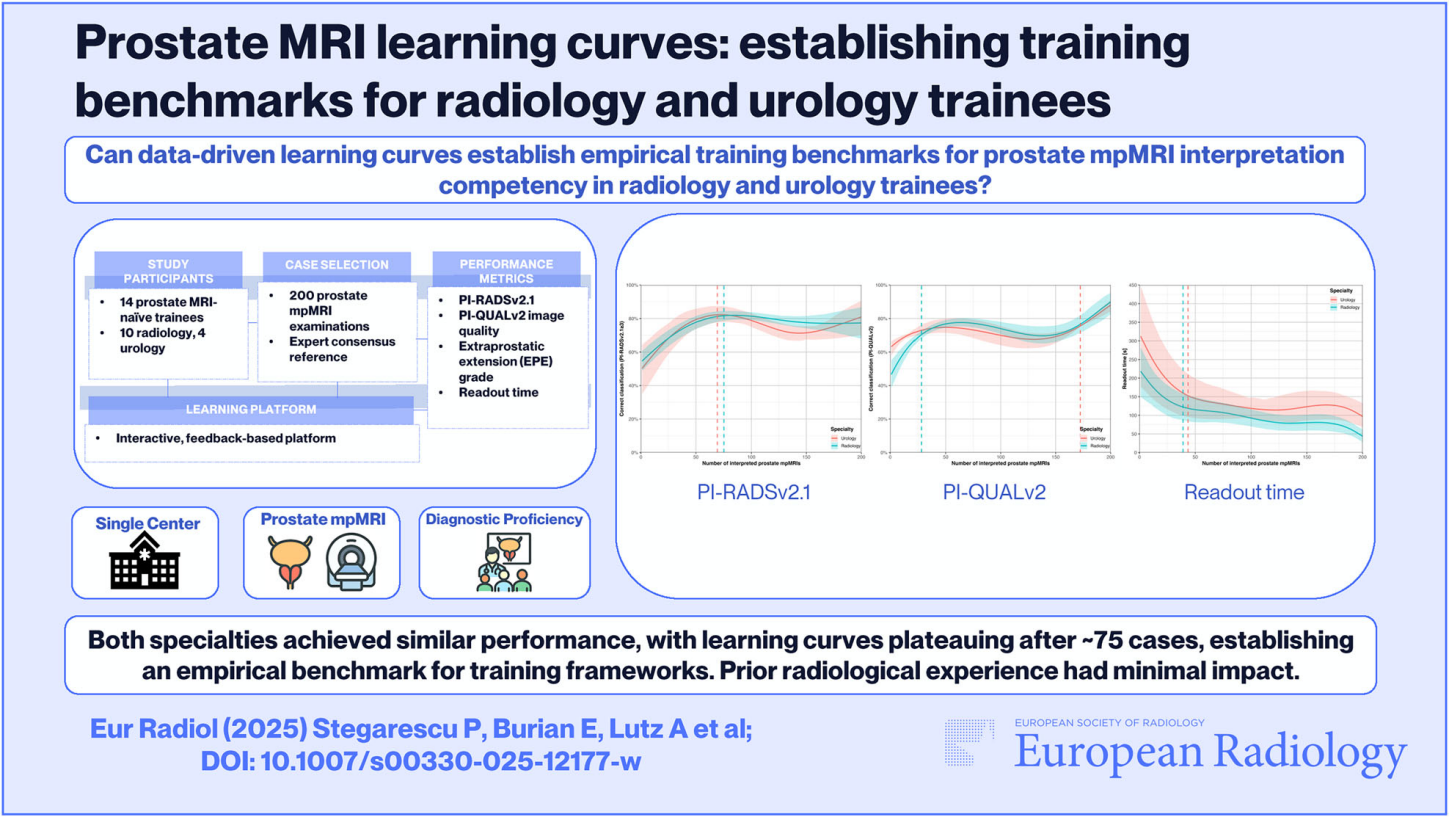

## Introduction

Multiparametric MRI (mpMRI) has become a cornerstone in prostate cancer diagnosis and management, with its integration into major clinical guidelines driving demand for high-quality interpretation [1–4]. To ensure diagnostic accuracy and reproducibility, radiological societies across Europe and North America have established or are developing certification frameworks that specify training requirements and case thresholds for readers [5–10]. However, these standards remain largely consensus-driven [11], lacking empirical benchmarks for optimal training volume, timing, duration, and competency.

Review of the prostate MRI education literature reveals critical methodological shortcomings. Most studies use small sample sizes and fixed-case block analyses [12–16], rather than true learning curves, failing to capture learning trajectories. Only one study used statistical modeling of learning curves to characterize early skill acquisition [17]. Prior research has focused mainly on lesion detection and localization [12, 15, 17], neglecting other interpretation domains such as staging and image quality assessment—now recognized as critical for interpretability and diagnostic accuracy [11, 18, 19]. Whether prior radiological experience alters training success also remains unexplored, leaving open critical questions: How many cases are required to achieve proficiency? What is the optimal timing for prostate MRI training—during residency or deferred to fellowship?

The clinical landscape is evolving beyond traditional radiological practice: urologists are increasingly reviewing prostate MRI for biopsy targeting and surgical planning, reflecting broader trends toward multidisciplinary imaging integration [20, 21]. Yet, despite this growing exposure, formal prostate mpMRI instruction is missing from most urology residency curricula, with only 17–23% of residents reporting structured training [21, 22]. The feasibility and effectiveness of such training, particularly for urologists without prior imaging experience, remain largely unexplored, and direct comparative data between specialties are scarce [23–25]. These evidence gaps leave current certification curricula for both radiologists and urologists without an empirical foundation.

We hypothesized that structured, feedback-based training would enable both radiology and urology trainees to achieve comparable performance in prostate mpMRI interpretation across key competencies— Prostate Imaging Reporting and Data System version 2.1 (PI-RADSv2.1) classification, Prostate Imaging Quality version 2 (PI-QUALv2) scoring, extraprostatic extension (EPE) grading, and readout time efficiency—independent of prior radiological experience. By modeling learning curves with flexible methods, we aimed to define empirical benchmarks for curriculum development, including case numbers required for routine-level reporting competency. Additionally, we quantified the influence of prior radiological experience to inform optimal timing of prostate mpMRI education.

## Materials and methods

### Study design and participants

Ethical approval was obtained from the Ethikkommission Ostschweiz (EKOS). Claude 4.0 Sonnet (Anthropic) was used for statistical code assistance and wording refinement; all outputs were verified by the authors, who assume full responsibility for the work. This prospective educational study was conducted at two hospitals between February and April 2025. Fourteen trainees participated, comprising ten radiology trainees with varying levels of radiological experience (nine residents, one fellow) and four urology residents, all without prior prostate mpMRI experience. At our institution, radiology residency is structured around subspecialty divisions (e.g., abdominal, cardiothoracic), so trainees accumulate MRI experience across multiple organ systems in line with their overall time in training, whereas urology residents receive no formal MRI instruction within their curriculum.

### MpMRI protocol

MpMRI scans included triplanar (axial, coronal, sagittal) T2-weighted imaging, diffusion-weighted imaging with apparent diffusion coefficient maps, and dynamic contrast-enhanced imaging. All scans were performed at 3 T without an endorectal coil. Detailed scanner specifications and sequence parameters are provided in Supplementary Tables 1–3.

### Case selection and reference standard

A total of 202 consecutive prostate mpMRI examinations performed between June and November 2024 were identified from the institutional PACS. Only men with no prior prostate cancer diagnosis were included, with no additional exclusion criteria applied, ensuring a representative clinical spectrum. Two introductory cases were used to familiarize participants with the platform and study workflow, leaving 200 cases for analysis. The reference standard was established via double expert consensus: initial report by certified prostate MRI radiologists [6], second review by certified uroradiologist (DD; fellowship trained, 7 years of prostate mpMRI experience, ~400 cases/year); disagreements resolved jointly. Extracted metrics: PI-RADSv2.1 score, lesion localization (T2 slice range), PI-QUALv2 image quality (three-point scale: 1–3) [18], and EPE grading (four-point scale: 0–3) [26].

### Learning platform and study workflow

Two weeks prior to study initiation, participants received instructional videos on platform use, expert readouts of the 2 training cases, and training materials [18, 26, 27]. All participants were asked to complete 200 study cases within 8 weeks. Cases were grouped into balanced sets of ten, maintaining the original PI-RADSv2.1 distribution to avoid clustering by case difficulty. All trainees interpreted the cases in the same predefined order. A custom platform enabled standardized entry of all scores and lesion locations (marked as a single T2 slice), with automatic recording of readout time. Participants reported one index lesion (highest PI-RADSv2.1 score); if multiple lesions shared the same highest score, any of those locations was considered correct. Cases were accessed via the institutional PACS using an anonymized list and standardized hanging protocol. For each case, patient age and PSA (if available) were provided. Embedded references (PI-QUALv2 publication [18], EPE grading graphic [28], and PI-RADSv2.1 educational resources [27]) were accessible throughout interpretation. Immediate feedback on answer correctness, cumulative performance, and graphical progress summaries was provided after each submission (see Supplementary Fig. 1).

### Statistical analysis

All analyses were performed in R (version 4.3.1). Primary outcome: agreement with reference standard for PI-RADSv2.1 ≥ 3 versus < 3 with correct lesion localization. Secondary outcomes: exact agreement for PI-RADSv2.1 (full 5-point scale), PI-QUALv2, EPE grading, readout time per case. Learning curves for each outcome were modeled using generalized estimating equations (GEE), with participant as the clustering variable to account for repeated measures. Fixed effects included trainee specialty (urology vs radiology), case number, and their interaction term to assess differences in learning rates. Odds ratios (ORs) are reported as urologists versus radiologists (i.e., OR > 1 indicates higher odds for urologists; OR < 1 indicates higher odds for radiologists). The optimal functional form for each trajectory (linear, logarithmic, square root, quadratic, cubic, or restricted cubic splines with up to five knots) was selected by minimizing a penalized quasi-likelihood information criterion (QIC): BIC-like-QIC = −2⋅Quasi Likelihood + log(*n*_clusters_)⋅*p*, where *n*_clusters_ represents the number of participants and *p* the number of model parameters. We performed separate GEE analyses including years of prior radiological experience (recorded as the exact number of days elapsed since the start of radiology residency and converted to decimal years) and case number as fixed effects, to examine the impact of prior imaging experience. Segmented regression was used to identify inflection points in learning curves, allowing one breakpoint per specialty, via iterative maximum likelihood estimation (convergence threshold: < 1 case change between iterations). This analytic framework was applied identically to all primary and secondary outcome measures. Separate GEE models were fitted to estimate sensitivity (proportion of positive reference cases correctly identified) and specificity (proportion of negative reference cases correctly identified) for the primary outcome. These estimates were used to compute Cohen’s κ for inter-rater agreement on binary PI-RADSv2.1 scores between trainees and the consensus reference. Inter-expert κ was calculated directly from the observed expert ratings across all cases. Additional analyses evaluated: (1) the fixed effect of image quality (PI-QUALv2) on exact PI-RADSv2.1 agreement by adding PI-QUALv2 as a covariate to various GEE models, and (2) changes in PI-RADSv2.1 category 3 assignment frequency with increasing case number experience by using generative additive models for plotting of smoothed proportions and fitting GEE models with case number as a linear predictor. Stratified cluster bootstrapping (1000 iterations, stratified by specialty and clustered by reader) was used to generate 95% confidence intervals for all key estimates.

## Results

### Patient and reader characteristics

Two hundred prostate mpMRI cases (median patient age 67.0 [interquartile range (IQR): 61.0, 72.0], median PSA 6.7 ng/mL [IQR: 5.0, 9.6], where available) were interpreted by 10 radiology trainees (median experience 2.7 years [IQR: 1.2, 3.9]) and 4 urology trainees (no prior imaging experience). PI-RADSv2.1 distribution, EPE grade distribution and details on participants are provided in Table 1.

### Inter-expert agreement

Reviewers disagreed in 47/200 cases (23.5%) for exact PI-RADSv2.1 (κ = 0.67 [95% CI: 0.60, 0.75]) and 38/200 (19.0%) for binary classification (≥ 3 vs < 3; κ = 0.63 [0.52, 0.73]); both indicate substantial agreement. Most discrepancies (85%) were downgrades on re-read; all were resolved by consensus.

### PI-RADSv2.1 classification performance

**Fig. 1:**
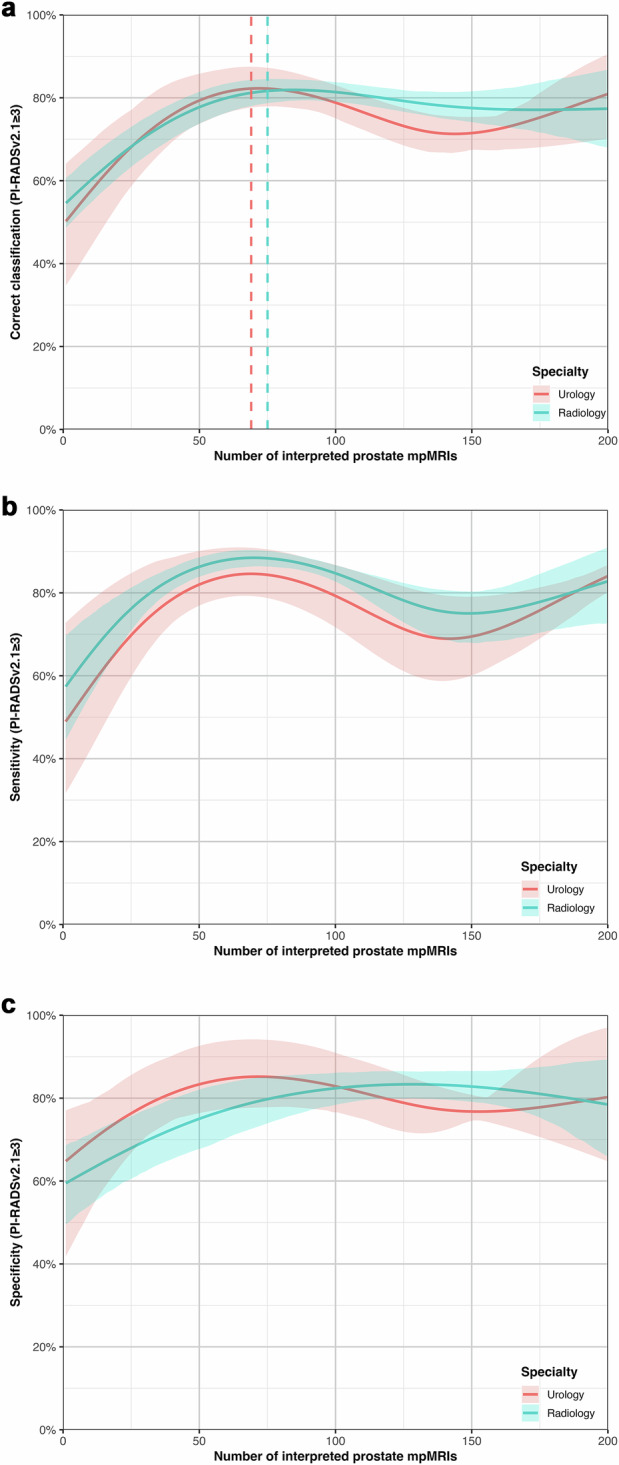
**a**–**c** Learning curves for the primary outcome of PI-RADSv2.1 classification (≥ 3 vs < 3) across 200 prostate multiparametric MRI cases showing performance trajectories for radiology and urology trainees. Fitted models: generalized estimating equations with cubic splines. **a** Overall percent agreement with the consensus reference standard. Dashed vertical lines indicate inflection points from segmented regression analysis. **b** Sensitivity learning curves showing proportion of positive reference cases correctly identified. **c** Specificity learning curves showing the proportion of negative reference cases correctly identified

A cubic spline GEE model with two internal knots provided the best fit for the learning curves (Supplementary Table 4, Fig. 1a–c). Prior radiological training was not associated with higher agreement in PI-RADSv2.1 assessment (OR per year 1.06, 95% CI: 0.96, 1.16). No significant difference in agreement with the reference standard was observed between specialties across 200 cases, except for cases 100–150, where radiology trainees outperformed urology trainees (OR 0.73; [0.54, 0.96]; Table 2). Both groups showed rapid improvement in the first 50 cases (urology trainees: +29.94% [21.33, 42.97]; radiology trainees: +23.77% [20.25, 28.51]). Segmented regression identified inflection points at 69 cases (46.0, 76.7) for urology trainees and 75 cases (64.0, 81.0) for radiology trainees. Beyond these points, no significant further improvement was observed (urology trainees: –1.31% [−11.2, 10.9]; radiology trainees: −4.29% [−12.8, 3.19]). Both groups demonstrated a transient decrease in agreement rates from cases 100–150 (urology trainees: −7.33% [−11.77, −4.00]; radiology trainees: −3.84% [−6.40, −1.48]). Final agreement with the reference standard for binary PI-RADSv2.1 (≥ 3 vs < 3) was 80.9% (72.4, 91.8) for urology trainees and 77.4% (68.5, 86.7) for radiology trainees (OR 1.24 [0.55, 3.10]). Learning curves for sensitivity and specificity (Fig. 1b, c) demonstrated similar patterns to overall agreement, with sensitivity curves showing more variable trajectories compared to specificity. Final sensitivity and specificity were 0.84 (0.78, 0.87) and 0.80 (0.64, 0.99) for urology trainees, and 0.83 (0.73, 0.91) and 0.79 (0.65, 0.90) for radiology trainees. Model-based final Cohen’s κ values showed substantial agreement with the reference standard for PI-RADSv2.1 ≥ 3 (κ = 0.64 [0.45, 0.82] for urology; κ = 0.61 [0.38, 0.77] for radiology). Exact PI-RADSv2.1 score agreement with the reference standard was 67.89% (55.52, 85.40) in urology trainees and 72.63% (61.61, 81.88) in radiology trainees (OR 0.80 [0.37, 2.23]). Supplementary Fig. 2 and Supplementary Table 5 show learning curve analyses for exact PI-RADSv2.1 score matching. Supplementary analyses on the impact of image quality (PI-QUALv2) on exact PI-RADSv2.1 agreement found no significant association (Supplementary Table 6). Additional analysis revealed significant decreases in PI-RADS 3 assignment probability with increasing experience: absolute reductions of 10.19% [95% CI: 5.77, 13.05] for radiology trainees and 4.16% [95% CI: 0.24, 9.87] for urology trainees across the 200-case training period (Supplementary Fig. 3).

### PI-QUALv2 image quality assessment

**Fig. 2:**
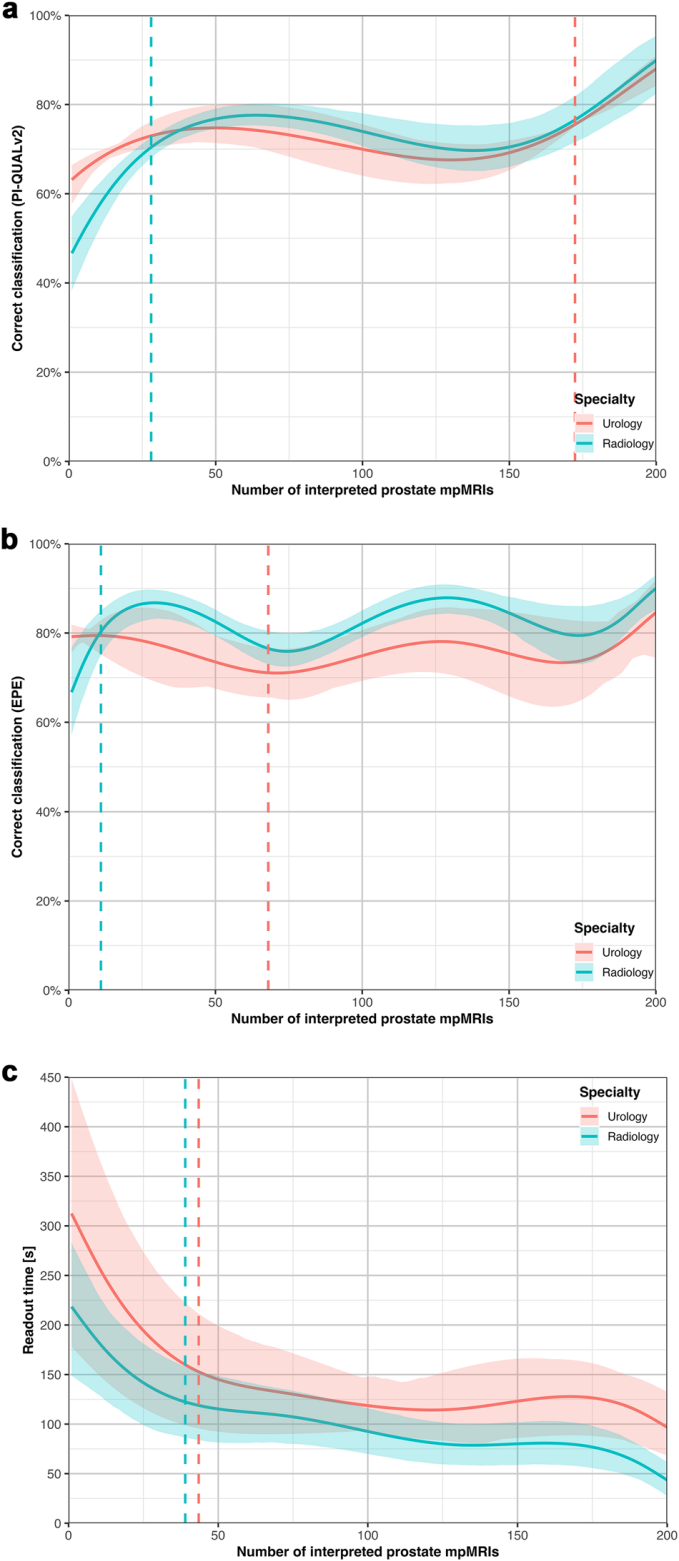
**a**–**c** Learning curves for secondary outcomes. Fitted models: generalized estimating equations with cubic polynomial (**a**) and cubic splines (**b**, **c**). **a** PI-QUALv2 image quality assessment. **b** Extraprostatic extension (EPE) grading assessment. **c** Case readout time efficiency. Dashed vertical lines indicate inflection points from segmented regression analysis. For **a** (both specialties) and **b** (urology trainees), segmented regression could not reliably identify single transition points, as indicated by wide confidence intervals

A cubic polynomial GEE model provided the best fit (Supplementary Table 7, Fig. 2a). Prior radiological experience had no significant impact on the agreement rate of PI-QUALv2 assessment (OR per year, 1.05 [0.99, 1.23]). Urology trainees demonstrated higher baseline agreement rates (62.54% [56.52, 65.96]) compared to radiology trainees (45.36% [36.86, 54.20]; OR 2.01 [1.35, 3.02], Table 3). Early learning (cases 0–50) was steeper for radiology trainees (+31.43% [21.70, 40.97]) compared to urology trainees (+12.21% [6.08, 23.70]), resulting in performance convergence by case 50 (agreement rate urology trainees: 74.76% [71.53, 80.37]; radiology trainees: 76.79%, [74.56, 78.91]; OR 0.90 [0.72, 1.22]). Both groups plateaued between cases 50–150, then improved again in the final segment (urology trainees: +18.80% [12.23, 25.60]; radiology trainees: +19.41% [9.53, 26.22]). Segmented regression revealed imprecise inflection points with wide confidence intervals (urology trainees: 172.35 [12.00, 177.00]; radiology trainees: 28 [25.00, 181.00]), indicating variable learning patterns without one single clearly defined transition point. Final agreement rates reached 88.0% (84.24, 90.87) for urology trainees and 89.89% (82.28, 95.27) for radiology trainees, with no significant difference between specialties (OR 0.82 [0.35, 1.72]).

### Extraprostatic extension (EPE) grading

A cubic spline GEE model with 4 internal knots provided the best fit (Supplementary Table 8, Fig. 2b). Prior radiological training had a small but significant impact on the agreement rates with the reference standard of EPE grading (OR per year 1.11 [1.03, 1.24]). At baseline, urology trainees demonstrated significantly higher agreement rates than radiology trainees (1.90 [1.11, 2.93], Table 4), but this was offset by a steeper early learning curve for radiology trainees in the first 50 cases (+17.57% [9.45, 28.78] vs −5.65% [−11.93, 4.47]; OR 0.59 [0.41, 0.85]). Radiology trainees showed sustained improvement throughout (+25.04% [15.18, 36.12]), while urology trainees’ performance remained stable, with no significant improvement observed by study end (+5.44% [−4.48, 17.50]). Segmented regression identified an early inflection point for radiology trainees at 11.0 cases (11.0, 13.9). No stable inflection point was identified for urology trainees (68.0; [26.0, 173.0]). Final agreement rates reached 84.59% (76.40, 93.18) for urology trainees and 90.0% (84.68, 92.75) for radiology trainees (OR 0.61 [0.31, 1.42]).

### Readout time

A cubic spline GEE model with four internal knots provided the best fit (Supplementary Table 9, Fig. 2c). Prior radiological training had no significant effect on readout time reduction (−6.85 s/year [−17.44, 5.57]). Mean readout times for urology trainees were consistently higher, but this difference was not statistically significant at any point (final segment difference: 53.26 s [−9.42, 95.88], Table 5). Both specialties demonstrated the most pronounced decrease in readout time in the first 50 cases (urology trainees: 167.5 s [76.40, 256.13]; radiology trainees: 101.35 s [38.51, 169.06]). Inflection points occurred at 43.47 cases (6.18, 64.85) for urology trainees and 39.00 cases (4.56, 83.00) for radiology trainees. By case 200, urology trainees had reduced their readout times by 215.75 s (71.74, 349.23) and radiology trainees by 174.01 s per case (113.08, 232.80).

**Table 1:** Patient and participant characteristics

Patient characteristics	
Number	200
Age (years) [IQR]	67.0 [61.0, 72.0]
PSA (ng/mL) [IQR]	6.7 [5.0, 9.6] (*n* = 177 available, *n* = 23 missing)
PI-RADSv2.1 scores (%)	
PI-RADS 1 (%)	4.0
PI-RADS 2 (%)	49.5
PI-RADS 3 (%)	4.5
PI-RADS 4 (%)	19.5
PI-RADS 5 (%)	22.5
PI-QUALv2 scores (%)	
PI-QUAL 1 (%)	1.0
PI-QUAL 2 (%)	28.5
PI-QUAL 3 (%)	70.5
EPE grades (%)	
EPE Grade 0 (%)	75.5
EPE Grade 1 (%)	11.5
EPE Grade 2 (%)	9.5
EPE Grade 3 (%)	3.5
Study participants	
Radiology trainees, *n*	10
PGY-1, *n* (%)	2 (20.0)
PGY-2, *n* (%)	2 (20.0)
PGY-3, *n* (%)	2 (20.0)
PGY-4, *n* (%)	1 (10.0)
PGY-5, *n* (%)	2 (20.0)
Fellow, *n* (%)	1 (10.0)
Median experience, years [IQR]	2.7 [1.2, 3.9]
Urology trainees, *n*	4
PGY-1, *n* (%)	1 (25.0)
PGY-2, *n* (%)	2 (50.0)
PGY-3, *n* (%)	1 (25.0)

*PI-RADSv2.1* Prostate Imaging Reporting and Data System version 2.1, *PI-QUALv2* Prostate Imaging Quality version 2, *EPE* extraprostatic extension, *IQR* interquartile range, *PGY* postgraduate year

**Table 2:** Learning curve metrics for PI-RADSv2.1 ≥ 3 classification

	Radiologists		Urologists		Comparison
Case number	Learning rate (% change per segment)	Cumulative improvement (% versus baseline)	Learning rate (% change per segment)	Cumulative improvement (% versus baseline)	OR (Uro vs Rad)
0	Baseline	Baseline	Baseline	Baseline	0.84 (0.42, 1.55)
0–50	23.77 (20.25, 28.51)*	23.77 (20.25, 28.51)*	29.94 (21.33, 42.97)*	29.94 (21.33, 42.97)*	1.10 (0.73, 1.89)
50–100	3.62 (1.35, 6.17)*	27.39 (23.04, 33.04)*	−0.53 (−3.02, 2.98)	29.41 (18.58, 43.22)*	0.85 (0.63, 1.23)
100–150	−3.84 (−6.40, −1.48)*	23.55 (18.47, 28.93)*	−7.33 (−11.77, −4.00)*	22.08 (11.70, 39.22)*	0.73 (0.54, 0.96)*
150–200	−0.17 (−7.73, 6.63)	23.39 (14.25, 32.37)*	9.44 (2.80, 19.23)*	31.52 (11.82, 58.46)*	1.24 (0.55, 3.10)

Data in parentheses are bootstrapped 95% confidence intervals

*PI-RADSv2.1* Prostate Imaging Reporting and Data System version 2.1, *OR* odds ratio

* 95% confidence interval does not include 1 (for OR) or 0 (for learning rate). Learning rates show absolute percentage change for each segment

**Table 3:** Learning curve metrics for PI-QUALv2 classification

	Radiologists		Urologists		Comparison
Case number	Learning rate (% change per segment)	Cumulative improvement (% versus baseline)	Learning rate (% change per segment)	Cumulative improvement (% versus baseline)	OR (Uro vs Rad)
0	Baseline	Baseline	Baseline	Baseline	2.01 (1.35, 3.02)*
0–50	31.43 (21.70, 40.97)*	31.43 (21.70, 40.97)*	12.21 (6.08, 23.70)*	12.21 (6.08, 23.70)*	0.90 (0.72, 1.22)
50–100	−2.81 (−5.92, 0.56)	28.61 (19.89, 37.11)*	−4.78 (−8.47, −1.53)	7.43 (−1.57, 19.00)	0.82 (0.58, 1.11)
100–150	−3.49 (−6.50, −0.57)*	25.12 (17.32, 33.32)*	−0.77 (−4.94, 1.60)	6.67 (0.14, 13.88)*	0.94 (0.67, 1.20)
150–200	19.41 (9.53, 26.22)*	44.54 (30.85, 57.64)*	18.80 (12.23, 25.60)*	25.46 (20.63, 33.26)*	0.82 (0.35, 1.72)

Data in parentheses are bootstrapped 95% confidence intervals

*PI-QUALv2* Prostate Imaging Quality version 2, *OR* odds ratio

* 95% confidence interval does not include 1 (for OR) or 0 (for learning rate). Learning rates show absolute percentage change for each segment

**Table 4:** Learning curve metrics for EPE grading

	Radiologists		Urologists		Comparison
Case number	Learning rate (% change per segment)	Cumulative improvement (% versus baseline)	Learning rate (% change per segment)	Cumulative improvement (% versus baseline)	OR (Uro vs Rad)
0	Baseline	Baseline	Baseline	Baseline	1.9 (1.11, 2.93)*
0–50	17.57 (9.45, 28.78)*	17.57 (9.45, 28.78)*	−5.65 (−11.93, 4.47)	−5.65 (−11.93, 4.47)	0.59 (0.41, 0.85)*
50–100	−0.35 (−3.22, 1.93)	17.22 (8.83, 27.46)*	1.42 (−1.87, 3.81)	−4.23 (−9.70, 5.52)	0.65 (0.46, 0.94)*
100–150	2.32 (−1.13, 5.45)	19.54 (12.36, 29.43)*	0.48 (−5.23, 4.08)	−3.75 (−13.57, 6.69)	0.56 (0.32, 1.01)
150–200	5.50 (−0.53, 10.58)	25.04 (15.18, 36.12)*	9.19 (−4.60, 20.55)	5.44 (−4.48, 17.50)	0.61 (0.31, 1.42)

Data in parentheses are bootstrapped 95% confidence intervals

*EPE* extraprostatic extension, *OR* odds ratio

* 95% confidence interval does not include 1 (for OR) or 0 (for learning rate). Learning rates show absolute percentage change for each segment

**Table 5:** Learning curve metrics for readout time

	Radiologists		Urologists		Comparison
Case number	Time saved versus segment baseline (s/case)	Time saved versus baseline (s/case)	Time saved versus segment baseline (s/case)	Time saved versus baseline (s/case)	Mean difference (urology vs radiology) (s/case)
0	Baseline	Baseline	Baseline	Baseline	94.03 (−79.90, 242.95)
0–50	101.35 (38.51, 169.06)*	101.35 (38.51, 169.06)*	167.51 (76.40, 256.13)*	167.51 (76.40, 256.13)*	29.82 (−58.27, 115.08)
50–100	22.11 (6.19, 41.91)*	124.88 (56.59, 188.46)*	26.34 (−18.11, 65.08)	193.85 (69.35, 303.21)*	26.07 (−27.85, 72.85)
100–150	12.44 (0.24, 24.70)*	137.13 (71.13, 203.07)*	−4.47 (−36.20, 20.19)	189.38 (28.05, 308.62)*	43.02 (−13.81, 106.41)
150–200	36.62 (20.21, 54.14)*	174.01 (113.08, 232.80)*	26.36 (−7.87, 61.92)	215.75 (71.74, 349.23)*	53.26 (−9.42, 95.88)

Data in parentheses are bootstrapped 95% confidence intervals

*OR* odds ratio

* 95% confidence interval does not include 0

## Discussion

Our study assessed learning curves of urology and radiology trainees interpreting 200 prostate mpMRI cases using a feedback-based platform across key competencies: PI-RADSv2.1 classification, PI-QUALv2 image quality scoring, EPE grading, and readout time. Learning trajectories for PI-RADSv2.1 classification and reporting speed were parallel across specialties, with major improvement within 75 cases, followed by plateaus. Radiology trainees started with lower performance for select metrics but rapidly closed gaps via steeper learning curves. Prior radiological experience minimally impacted learning or final performance, except for a small significant effect on EPE grading. By training end, no significant performance differences remained between groups across all metrics.

Earlier studies provided insights into prostate MRI learning before PI-RADS standardization and without trajectory modeling [12, 13, 16]. Salka et al assessed the PPV in 50-case blocks for PI-RADSv2 3–5 lesions interpreted by eight experienced radiologists, focusing on long-term skill acquisition rather than initial learning dynamics [15]. Rosenkrantz et al provided the only comprehensive PI-RADSv2-based learning curve analysis of six radiology residents interpreting 124 cases [17]. Consistent with our findings, they observed rapid early improvement followed by a plateau. Our study expands prior work by analyzing a contemporary cohort using PI-RADSv2.1 and additional clinically relevant metrics, enabling comprehensive interpretive skill assessment. Unlike previous works [17], we avoided a priori assumptions about learning curve shapes, finding that flexible non-linear approaches best captured learning pattern variability. Our biopsy-naïve cohort reflects contemporary practice with relaxed MRI indications, while Rosenkrantz et al included men with prior negative biopsies or known cancer, likely contributing to later inflection points (69–75 vs 40 cases [17]).

Despite comparable performance, some specialty differences emerged. For PI-QUALv2 assessment, urology trainees outperformed radiology trainees at baseline (62.5% vs 43.7%) despite lacking formal radiological training, suggesting PI-QUALv2 is intuitive for clinicians without an imaging background. This gap was rapidly closed by radiology trainees’ steeper learning curves within 50 cases, aligning with studies showing single lectures can improve PI-QUAL scoring across specialties [29]. Similar final performance between specialties for all metrics challenges assumptions that extensive radiological training is a prerequisite for prostate mpMRI interpretation. Prior radiological experience showed no significant effect on PI-RADSv2.1 classification (OR per year 1.06 [0.96, 1.16]). Even at the upper CI bound (OR 1.16), potential benefit remains modest, ~16% increased rates of agreement per year versus 30% improvement through structured training in the first 50 cases. EPE grading showed different patterns with a small experience effect, yet both groups achieved similar final agreement rates.

These findings have important implications for curriculum design and timing. The minimal impact of prior radiological experience indicates that prostate mpMRI interpretation skills can be acquired by trainees from different specialties early during residency training, rather than being restricted to fellowship-level instruction as is current practice in many institutions. While previous work showed the feasibility of training urologists in prostate mpMRI interpretation [23, 25], our study is the first to map and compare full learning curves across specialties, thereby providing an empirical curriculum foundation for both radiology and urology training.

The need for such programs is underscored by recent survey data highlighting significant educational gaps in urology training. In a Canadian–US cohort, only 12 of 53 urology residents (23%) reported receiving formal prostate mpMRI training, with poor understanding of PI-RADS scoring components [21]. Similarly, an international survey of 304 residents from Italy, Brazil, and the UK found that just 17–20% had received structured education and < 20% felt confident in prostate mpMRI interpretation, despite more than 90% expressing interest [22].

Standardized training across specialties offers tangible clinical benefits for urological practice. When urologists can accurately interpret imaging findings, they make more informed clinical decisions on biopsy targeting and surgical management while reducing the need for clarifications and communication delays with radiologists. Given that urologists will continue to encounter prostate mpMRI during routine care despite limited formal preparation, developing structured programs that complement, rather than replace, radiology expertise is warranted and is likely to translate into better patient care.

Our observed inflection points at 69–75 cases align with international certification frameworks and provide empirical support for training benchmarks. Programs in Germany [6], Austria [5], Europe [7], and the US [10] reflect growing interest, alongside emerging initiatives in the UK [9] and Canada [8]. German and Austrian radiological societies mandate 50 supervised cases, while the new ACR program recommends 100–150. By the training end, both specialties achieved agreement rates of 77.4–80.9% for PI-RADSv2.1 (≥ 3), 88.0–89.9% for PI-QUALv2, and 84.6–90.0% for EPE grading. For PI-RADSv2.1 ≥ 3, this translated into substantial agreement with the expert reference (Cohen’s κ = 0.64 for urology, 0.61 for radiology), matching the pooled κ of 0.57 (95% CI: 0.25–0.89) from a recent meta-analysis [30] and our inter-expert agreement (κ = 0.63). [30]. This alignment with expert-level agreement and published standards suggests that, after approximately 75 cases, trainees reach a competency level appropriate for supervised reporting. It is important to note, however, that our study evaluated routine cases only and did not test performance on complex pathologies or incidental extraprostatic findings (e.g., incidental rectal cancer), where broader radiological expertise is likely advantageous. Therefore, while our findings support readiness for supervised prostate mpMRI reporting, they do not indicate expert-level proficiency.

Limitations include: First, participant numbers were modest, particularly among urology trainees (*n* = 4 vs *n* = 10), resulting in wider confidence intervals for group comparisons. However, combining all 2800 observations per outcome in a single comprehensive GEE model enabled robust estimation of group-level learning trajectories despite limited cluster size; larger multicenter cohorts are needed for broader generalizability. Second, our reference standard relied on expert consensus rather than histopathology [15, 17], reflecting our educational focus on imaging criteria application; moreover, many negative or low-suspicion cases in clinical practice are not biopsied. Third, all trainees interpreted the same difficulty-balanced case sequence to ensure valid between-group comparisons; residual case-level variability may still have affected individual data points and curve shape. Fourth, slice-range localization may have overestimated lesion-level agreement. Fifth, limited positive EPE cases (24.5% prevalence) constrained learning opportunities, suggesting training curricula should supplement routine cases with enriched datasets [13]. Finally, our learning platform may not fully replicate real-world clinical complexity, and we did not address long-term skill retention; therefore, the durability of the observed plateau remains unknown.

In conclusion, our study establishes an empirical benchmark of ~75 cases for prostate mpMRI competency, achievable early in training by both radiology and urology trainees, regardless of prior imaging experience. As urologists are increasingly exposed to prostate mpMRI without formal training, we present a feasible training pathway intended to complement, not replace, the primary diagnostic role of radiologists, and to foster more effective interdisciplinary care. Further research should assess long-term skill retention, clinical outcomes, and broader diagnostic responsibilities.

## Supplementary information

ELECTRONIC SUPPLEMENTARY MATERIAL

## Abbreviations

CI: Confidence interval
EPE: Extraprostatic extension
GEE: Generalized estimating equations
IQR: Interquartile range
mpMRI: Multiparametric MRI
OR: Odds ratio
PI-QUALv2: Prostate Imaging Quality version 2
PI-RADSv2.1: Prostate Imaging Reporting and Data System version 2.1

## References

[1] Moses KA, Sprenkle PC, Bahler C et al (2023) NCCN Guidelines® Insights: Prostate Cancer Early Detection, Version 1.2023. J Natl Compr Canc Netw 21:236–246 10.6004/jnccn.2023.0014 36898362

[2] Cornford P, Bergh RCN, Briers E (2023) EANM-ESTRO-ESUR-ISUP-SIOG guidelines on prostate cancer. In: EAU guidelines presented at the 2023 EAU Annual Congress, Milan. EAU Guidelines Office, Arnhem

[3] Expert Panel on Urological Imaging, Akin O, Woo S et al (2023) ACR Appropriateness Criteria® pretreatment detection, surveillance, and staging of prostate cancer: 2022 update. J Am Coll Radiol 20:S187–S210. 10.1016/j.jacr.2023.02.010 37236742 10.1016/j.jacr.2023.02.010

[4] Wei JT, Barocas D, Carlsson S et al (2023) Early detection of prostate cancer: AUA/SUO guideline Part I: Prostate cancer screening. J Urol 210:46. 10.1097/JU.0000000000003491 37096582 10.1097/JU.0000000000003491 PMC11060750

[5] Österreichische Röntgengesellschaft (n.d.) ÖRG-Quality-Assurance für die Durchführung und Befundung von mpMRTs der Prostata. ÖRG. https://www.oerg.at/quality-assurance. Accessed 13 May 2025

[6] Zertifizierung MRT der Prostata (n.d.) AG Uroradiologie in der Deutschen Röntgengesellschaft. https://www.ag-uro.drg.de. Accessed 25 May 2025

[7] European Certification in Prostate MRI (n.d.) ESUR. https://www.esur.org/european-certification-in-prostate-mri. Accessed 25 May 2025

[8] Chang SD, Reinhold C, Kirkpatrick IDC et al (2022) Canadian Association of Radiologists prostate MRI white paper. Can Assoc Radiol J 73:626–638. 10.1177/08465371221105532 35971326 10.1177/08465371221105532

[9] Barrett T, Padhani AR, Patel A et al (2021) Certification in reporting multiparametric magnetic resonance imaging of the prostate: recommendations of a UK consensus meeting. BJU Int 127:304–306. 10.1111/bju.15285 33113258 10.1111/bju.15285

[10] American College of Radiology (2025) Prostate cancer MRI center designation (2-27-2025). In: Accreditation support. Available via https://accreditationsupport.acr.org/support/solutions/articles/11000112416-prostate-cancer-mri-center-designation-2-27-2025-. Accessed 11 May 2025

[11] de Rooij M, Israël B, Tummers M et al (2020) ESUR/ESUI consensus statements on multi-parametric MRI for the detection of clinically significant prostate cancer: quality requirements for image acquisition, interpretation and radiologists’ training. Eur Radiol 30:5404–5416 32424596 10.1007/s00330-020-06929-z PMC7476997

[12] Gaziev G, Wadhwa K, Barrett T et al (2016) Defining the learning curve for multiparametric magnetic resonance imaging (MRI) of the prostate using MRI-transrectal ultrasonography (TRUS) fusion-guided transperineal prostate biopsies as a validation tool. BJU Int 117:80–86. 10.1111/bju.12892 25099182 10.1111/bju.12892

[13] Akin O, Riedl CC, Ishill NM et al (2010) Interactive dedicated training curriculum improves accuracy in the interpretation of MR imaging of prostate cancer. Eur Radiol 20:995–1002. 10.1007/s00330-009-1625-x 19921205 10.1007/s00330-009-1625-x PMC3609714

[14] Latchamsetty K, Borden L, Porter C et al (2007) Experience improves staging accuracy of endorectal magnetic resonance imaging in prostate cancer: what is the learning curve? Can J Urol 14:3429–3434 17324322

[15] Salka BR, Shankar PR, Troost JP et al (2022) Effect of prostate MRI interpretation experience on PPV using PI-RADS version 2: A 6-year assessment among eight fellowship-trained radiologists. AJR Am J Roentgenol 219:453–460. 10.2214/AJR.22.27421 35319914 10.2214/AJR.22.27421 PMC10170485

[16] Harris RD, Schned AR, Heaney JA (1995) Staging of prostate cancer with endorectal MR imaging: lessons from a learning curve. Radiographics 15:813–829. 10.1148/radiographics.15.4.7569131 7569131 10.1148/radiographics.15.4.7569131

[17] Rosenkrantz AB, Ayoola A, Hoffman D et al (2017) The learning curve in prostate MRI interpretation: self-directed learning versus continual reader feedback. AJR Am J Roentgenol 208:W92–W100 10.2214/AJR.16.16876 28026201

[18] De Rooij M, Allen C, Twilt JJ et al (2024) PI-QUAL version 2: an update of a standardised scoring system for the assessment of image quality of prostate MRI. Eur Radiol. 10.1007/s00330-024-10795-4 10.1007/s00330-024-10795-4 PMC11519155 38787428

[19] Brembilla G, Lavalle S, Parry T et al (2023) Impact of prostate imaging quality (PI-QUAL) score on the detection of clinically significant prostate cancer at biopsy. Eur J Radiol. 10.1016/j.ejrad.2023.110849 10.1016/j.ejrad.2023.110849 37141845

[20] Miszewski K, Skrobisz K, Miszewska L, Matuszewski M (2024) Interpreting prostate MRI reports in the era of increasing prostate MRI utilization: a urologist’s perspective. Diagnostics (Basel) 14:1060 10.3390/diagnostics14101060 PMC11120165 38786358

[21] Rodrigues C, Visram K, Sedghi A et al (2021) Attitudes and experience of urology trainees in interpreting prostate magnetic resonance imaging. Can Urol Assoc J 15:E293–E298. 10.5489/cuaj.6614 33119496 10.5489/cuaj.6614 PMC8095287

[22] Ippoliti S, Orecchia L, Esperto F et al (2023) Survey on prostate MRI reading and interpretation among urology residents in Italy, Brazil and the UK: a cry for help. Minerva Urol Nephrol 75:297–307. 10.23736/S2724-6051.22.05043-1 36286400 10.23736/S2724-6051.22.05043-1

[23] Kasivisvanathan V, Ambrosi A, Giganti F et al (2019) A dedicated prostate MRI teaching course improves the ability of the urologist to interpret clinically significant prostate cancer on multiparametric MRI. Eur Urol 75:203–204 30327275 10.1016/j.eururo.2018.09.033

[24] Mantica G, Suardi N, Smelzo S et al (2022) Are urologists ready for interpretation of multiparametric MRI findings? A prospective multicentric evaluation. Diagnostics (Basel) 12:2656. 10.3390/diagnostics12112656 36359499 10.3390/diagnostics12112656 PMC9689928

[25] Wang NN, Fan RE, Ghanouni P, Sonn GA (2019) Teaching urologists “how to read multi-parametric prostate MRIs using PIRADSv2”: results of an iBook pilot study. Urology 131:40–45. 10.1016/j.urology.2019.04.040 31150691 10.1016/j.urology.2019.04.040

[26] Mehralivand S, Shih JH, Harmon S et al (2019) A grading system for the assessment of risk of extraprostatic extension of prostate cancer at multiparametric MRI. Radiology 290:709–719. 10.1148/radiol.2018181278 30667329 10.1148/radiol.2018181278 PMC6394788

[27] Prostate cancer—PI-RADS v2.1. In: The radiology assistant. https://radiologyassistant.nl/abdomen/prostate/prostate-cancer-pi-rads-v2-1

[28] Park KJ, Kim M-H, Kim JK (2020) Extraprostatic tumor extension: comparison of preoperative multiparametric MRI criteria and histopathologic correlation after radical prostatectomy. Radiology 296:87–95. 10.1148/radiol.2020192133 32368959 10.1148/radiol.2020192133

[29] Giganti F, Cole A, Fennessy F et al (2022) Promoting the use of the PI-QUAL score for prostate MRI quality: results from the ESOR Nicholas Gourtsoyiannis teaching fellowship. Eur Radiol 33:461–471 35771247 10.1007/s00330-022-08947-5 PMC9244244

[30] Park KJ, Choi SH, Lee JS et al (2020) Interreader agreement with Prostate Imaging Reporting and Data System version 2 for prostate cancer detection: a systematic review and meta-analysis. J Urol. 10.1097/JU.0000000000001200 10.1097/JU.0000000000001200 32552474

