# Supplementary material for: Prostate MRI learning curves: establishing training benchmarks for radiology and urology trainees
Source: Eur Radiol. 2025 Dec 16;36(5):3431–41. doi: 10.1007/s00330-025-12177-w (PMC13086796; doi:10.1007/s00330-025-12177-w)

SUPPLEMENTAL MATERIAL

Supplemental Table 1. Overview of multiparametric MRI protocols.

Protocol No.	Magnet strength	Scanner Name	Coil type	T2 planes	DWI (b values)	DCE resolution(s), time points (n)
1	3T	Siemens MAGNETOM Vida	No ER	Triplanar	100, 800, 1500**	5, 45
2	3T	Philips Achieva dStream	No ER	Triplanar	100, 950, 1500	6, 15

*extrapolated. Abbreviations: DCE, Dynamic contrast enhancement; DWI, Diffusion-weighted imaging; ER, endorectal coil.

Supplemental Table 2. Sequence parameters for multiparametric MRI protocol 1

Parameter	T2w TSE (axial)	T2w TSE (coronal)	T2w TSE (sagittal)	DWI (axial)	DCE (axial)
TR/TE (ms)	7880/127	7620/148	7710/147	5100/68	3.92/1.68
Flip angle	160	160	160	90	18
Slice thickness (mm)	3	3	3	3	3
FOV (mm)	200x200	200x200	200x200	101x199	239x239
Acquisition Matrix	432 x 320	400 x 400	400 x 400	114 × 58	224 × 224

Abbreviations: DCE, Dynamic contrast enhancement; DWI, Diffusion-weighted imaging; FOV, Field of View; TE, Echo Time; TR, Repetition Time; TSE, Turbo Spin Echo.

Supplemental Table 3. Sequence parameters for multiparametric MRI protocol 2

Parameter	T2w TSE (axial)	T2w TSE (coronal)	T2w TSE (sagittal)	DWI (axial)	DCE (axial)
TR/TE (ms)	3126/100	3000/100	3000/100	4009/82.4	4.82/2.34
Flip angle	90	90	90	90	15
Slice thickness (mm)	3	3	3	3	4
FOV (mm)	180 x 180	180 x 180	180 x 180	180 x 180	180 x 180
Acquisition Matrix	400 x 384	360 x 332	300 x 295	84 x 81	180 x 180

Abbreviations: DCE, Dynamic contrast enhancement; DWI, Diffusion-weighted imaging; FOV, Field of View; TE, Echo Time; TR, Repetition Time; TSE, Turbo Spin Echo.

Supplemental Table 4. Model comparison for PI-RADSv2.1 ≥3 Classification

Model	QIC	ΔQIC	QICu	ΔQICu	BIC-like QIC	ΔBIC-like QIC
ns(case_num, knots=Q33,67)	3,060	6.89	3,051	2.37	3,056	0.000
ns(case_num, knots=Q5,35,65,95)	3,053	0.00	3,049	0.00	3,056	0.191
ns(case_num, knots=Q10,50,90)	3,059	6.50	3,052	3.02	3,058	1.929
Cubic Polynomial	3,065	11.71	3,056	7.10	3,061	4.738
ns(case_num, knots=Q5,27.5,50,72.5,95)	3,057	4.27	3,052	3.39	3,061	4.861
ns(case_num, knots=Q50)	3,074	20.62	3,063	14.81	3,067	11.162
Quadratic	3,075	22.51	3,065	16.74	3,069	13.099
Logarithmic	3,085	31.79	3,074	24.99	3,076	20.067
Square Root	3,092	38.97	3,079	30.62	3,082	25.699
Linear	3,101	47.79	3,087	38.76	3,090	33.836
Exponential	3,098	45.08	3,093	44.13	3,095	39.207

Qx = xth percentile of case_num.

Supplemental Table 5. Learning curve metrics for exact PI-RADSv2.1 classification

	Radiologists		Urologists		Comparison
**Case Number**	**Learning Rate (% change per segment)**	**Cumulative Improvement (% versus Baseline)**	**Learning Rate (% change per segment)**	**Cumulative Improvement (% versus Baseline)**	**OR (Uro vs Rad)**
0	Baseline	Baseline	Baseline	Baseline	0.68 (0.43, 1.17)
0-50	23.16 (16.01, 31.41)*	23.16 (16.01, 31.41)*	33.77 (25.93, 44.62)*	33.77 (25.93, 44.62)*	1.07 (0.56, 1.80)
50-100	10.18 (6.98, 14.17)*	33.34 (26.51, 41.41)*	5.23 (0.76, 12.73)*	39.00 (34.41, 48.35)*	0.86 (0.60, 1.17)
100-150	1.34 (-2.34, 4.50)	34.67 (29.84, 40.14)*	-5.88 (-10.91, -1.21)*	33.12 (25.41, 41.26)*	0.62 (0.48, 0.79)
150-200	-0.12 (-9.14, 5.20)	34.55 (26.26, 41.69)*	5.60 (-5.56, 19.08)	38.72 (27.23, 67.67)*	0.80 (0.37, 2.23)

Data in parentheses are bootstrapped 95% CIs. * 95% CI does not include 1 (for OR) or 0 (for learning rate/cumulative improvement).

Supplemental Table 6: Impact of image quality (PI-QUALv2) on exact PI-RADSv2.1 Agreement: Comparison of GEE models for exact PI-RADSv2.1 classification with PI-QUALv2 predictor effects

Model Nr.	Model Formula	BIC-like QIC	PI-QUAL_expert OR (95% CI)
**6**	**PIRADS_exact ~ ns(case_number, knots=Q33,67) + PI-QUAL_expert*specialty**	**3,547.0**	**1.012 (0.891–1.182)**
5	PIRADS_exact ~ ns(case_number, knots=Q33,67) * specialty + PI-QUAL_expert	3,548.0	1.059 (0.948–1.189)
2	PIRADS_exact ~ ns(case_number, knots=Q33,67) + PI-QUAL_expert	3,548.1	1.058 (0.948–1.188)
7	PIRADS_exact ~ ns(case_number, knots=Q33,67) * specialty + ns(case_number, knots=Q33,67) * PI-QUAL_expert	3,552.5	0.753 (0.572–1.111)
4	PIRADS_exact ~ PI-QUAL_expert * ns(case_number, knots=Q33,67)	3,552.6	0.753 (0.571–1.111)
3	PIRADS_exact ~ PI-QUAL_expert*specialty	3,647.9	1.006 (0.881–1.173)
1	PIRADS_exact ~ PI-QUAL_expert	3,648.7	1.046 (0.931–1.175)

Note: Models are ordered by QBIC (lower values indicate better fit). The best-fitting model is highlighted in blue. Bootstrap confidence intervals were calculated using 1000 bootstrap replicates per model. Q33,67 refers to quantile-based knots at the 33rd and 67th percentiles of case_number.

Interpretation:

• Best-fitting model: Model Nr. 6 (QBIC = 3547.0) includes learning curve transformation plus PI-QUAL_expert×specialty interaction

• Simple additive models (model nr. 2,3,5) show small positive PI-QUAL_expert associations (OR ≈ 1.06) with confidence intervals spanning 1.0 (i.e., not statistically significant)

• Interaction models with interaction terms case_numerxPI-QUAL_expert (models 4, 7) show different effect sizes (OR ≈ 0.75) with wider confidence intervals, suggesting PI-QUAL_expert effects vary across the learning curve

Supplemental Table 7. Model comparison for PI-QUALv2 Classification

Model	QIC	ΔQIC	QICu	ΔQICu	BIC-like QIC	ΔBIC-like QIC
Cubic Polynomial	3,220	0.443	3,220	0.00	3,225	0.00
ns(case_num, knots=Q10,50,90)	3,219	0.000	3,223	2.75	3,229	4.03
ns(case_num, knots=Q33,67)	3,226	6.600	3,226	5.98	3,231	5.98
ns(case_num, knots=Q5,35,65,95)	3,220	0.312	3,224	3.68	3,231	6.24
ns(case_num, knots=Q5,27.5,50,72.5,95)	3,220	0.560	3,226	5.93	3,235	9.76
Logarithmic	3,245	25.272	3,244	24.02	3,247	21.46
Square Root	3,251	31.868	3,251	30.53	3,253	27.97
Linear	3,256	36.408	3,255	34.97	3,258	32.41
Quadratic	3,256	36.857	3,256	36.41	3,260	35.13
ns(case_num, knots=Q50)	3,256	37.080	3,257	36.48	3,260	35.20
Exponential	3,268	48.897	3,270	50.43	3,273	47.88

Qx = xth percentile of case_num.

Supplemental Table 8. Model comparison for EPE Classification

Model	QIC	ΔQIC	QICu	ΔQICu	BIC-like QIC	ΔBIC-like QIC
ns(case_num, knots=Q5,35,65,95)	2,740	0.0000	2,740	0.00	2,748	0.00
ns(case_num, knots=Q5,27.5,50,72.5,95)	2,740	0.0458	2,741	1.01	2,750	2.29
Logarithmic	2,756	16.1047	2,748	8.14	2,751	3.03
Exponential	2,755	14.8680	2,749	9.17	2,752	4.06
Square Root	2,758	17.9382	2,750	9.82	2,753	4.70
Linear	2,759	18.4831	2,751	10.31	2,753	5.19
Quadratic	2,759	18.6135	2,753	12.92	2,757	9.08
ns(case_num, knots=Q50)	2,759	18.7950	2,753	13.08	2,757	9.24
ns(case_num, knots=Q33,67)	2,759	19.0964	2,756	15.62	2,761	13.06
Cubic Polynomial	2,760	20.0306	2,757	16.70	2,762	14.15
ns(case_num, knots=Q10,50,90)	2,761	20.3630	2,760	19.65	2,766	18.38

Qx = xth percentile of case_num.

Supplemental Table 9. Model comparison for readout times

Model	QIC	ΔQIC	QICu	ΔQICu	BIC-like QIC	ΔBIC-like QIC
Cubic Splines (4 knots)	17,689,557	0	17,689,557	0	17,689,227	0
Cubic Splines (5 knots)	17,693,775	4,218	17,693,775	4,218	17,693,447	4,220
Cubic Splines (3 knots)	17,778,076	88,520	17,778,076	88,520	17,777,747	88,519
Cubic Polynomial	17,781,909	92,352	17,781,576	92,019	17,781,581	92,354
Logarithmic	17,868,645	179,088	17,868,331	178,775	17,868,334	179,107
Cubic Splines (2 knots)	17,874,697	185,141	17,874,697	185,141	17,874,373	185,146
Quadratic	18,118,749	429,192	18,118,429	428,872	18,118,433	429,205
Cubic Splines (1 knot)	18,137,376	447,819	18,137,376	447,819	18,137,061	447,834
Square Root	18,287,089	597,533	18,286,789	597,232	18,286,791	597,564
Linear	18,841,127	1,151,570	18,840,848	1,151,292	18,840,851	1,151,624
Exponential	21,530,991	3,841,434	21,540,073	3,850,517	21,540,076	3,850,849

Qx = xth percentile of case_num.

Supplemental Figure 1. Custom made prostate mpMRI learning tool.

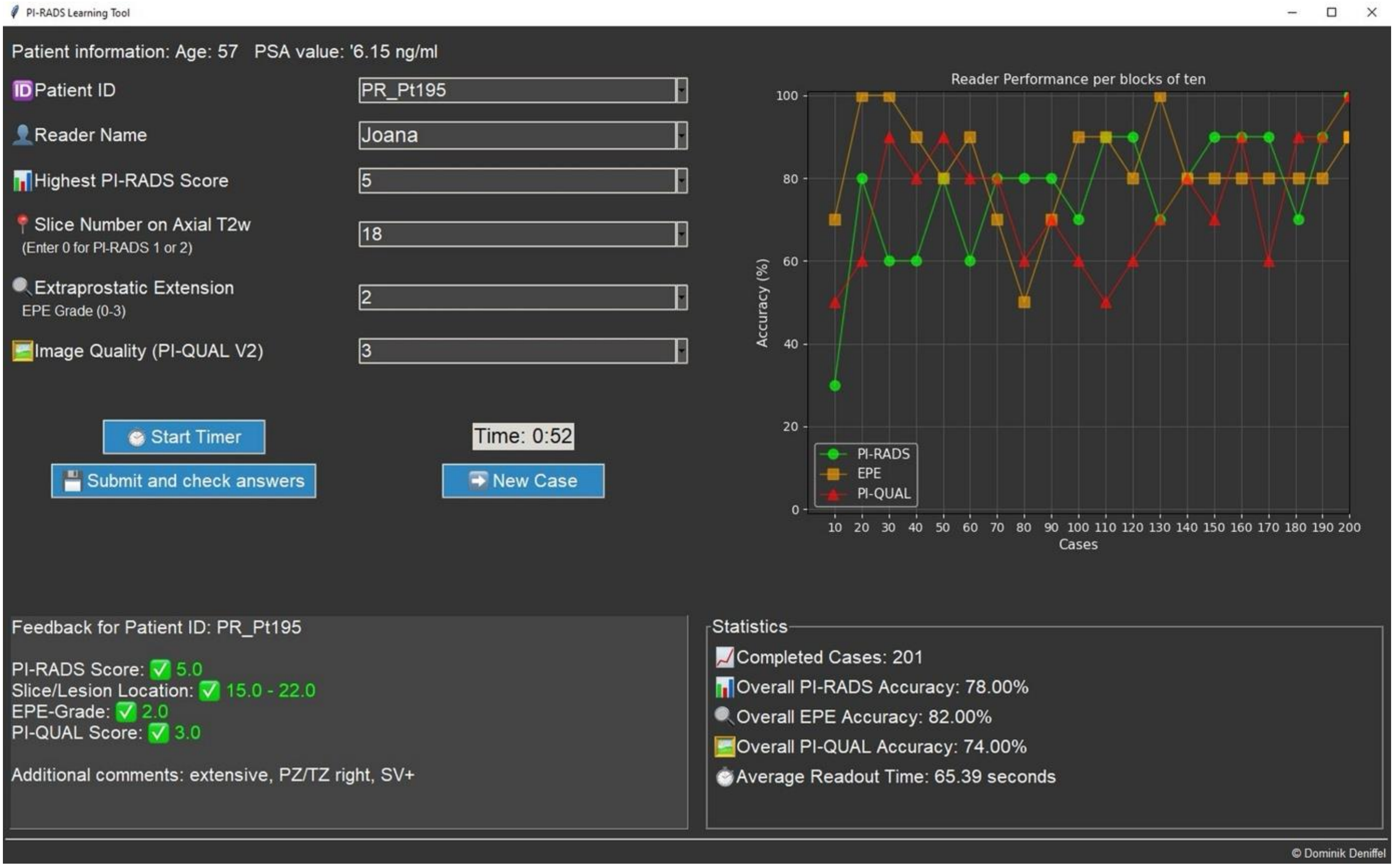

Supplemental Figure 2. Learning curve for exact PI-RADSv2.1 classification. Dashed vertical lines indicate inflection points from segmented regression analysis. Urology trainees: 71.00 cases (40.00, 72.00); radiology trainees: 77.91 cases (75.00, 118.00)

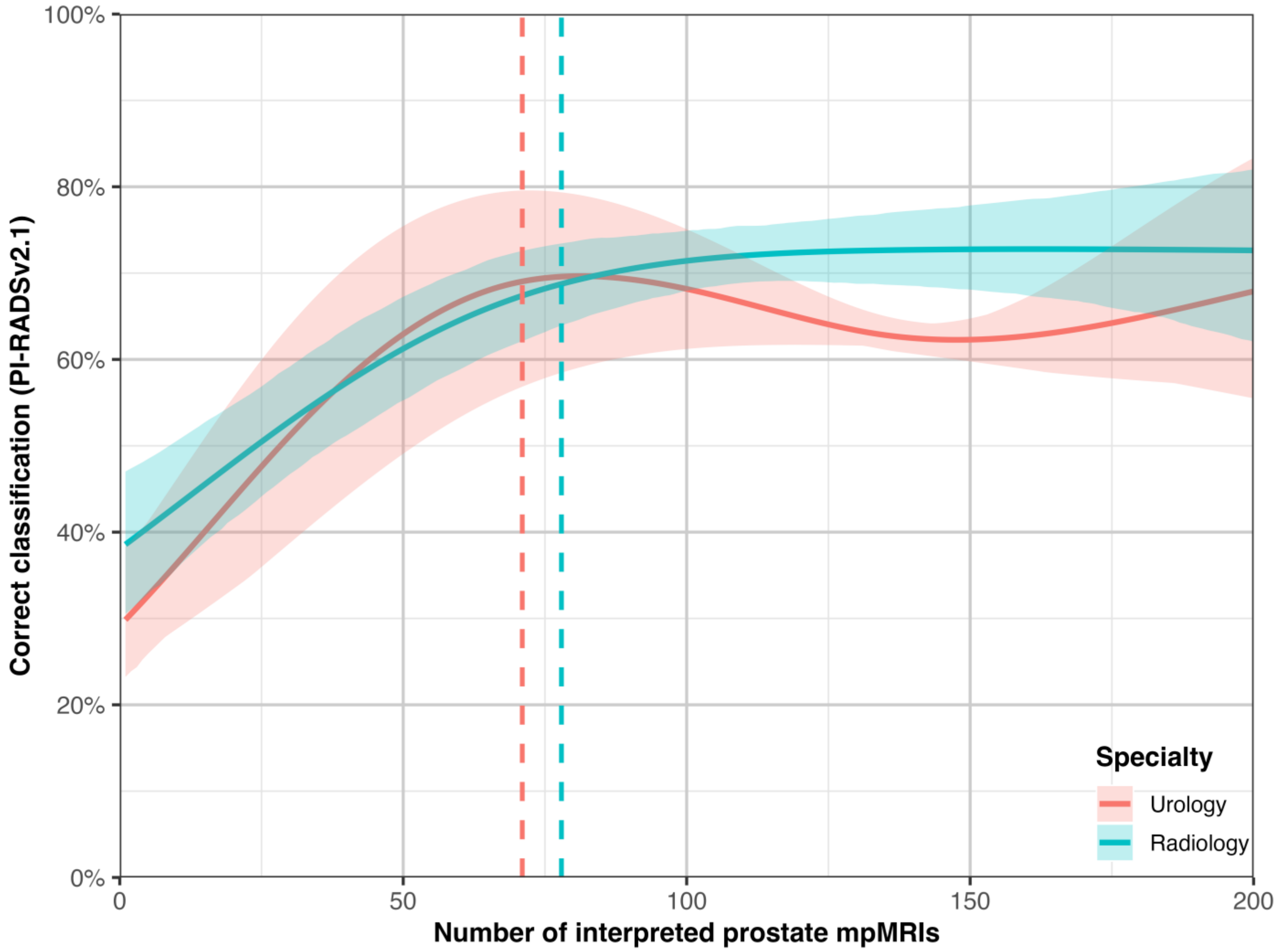

Supplemental Figure 3: PI-RADS 3 assignment rate by cumulative interpreted mpMRI case number.

Estimated PI-RADSv2.1 category 3 assignment rates from generalized additive models (GAM) with factor-by smooths for specialty. Vertical ticks demonstrate individual cases with PI-RADSv2.1 score 3 according to expert consensus.

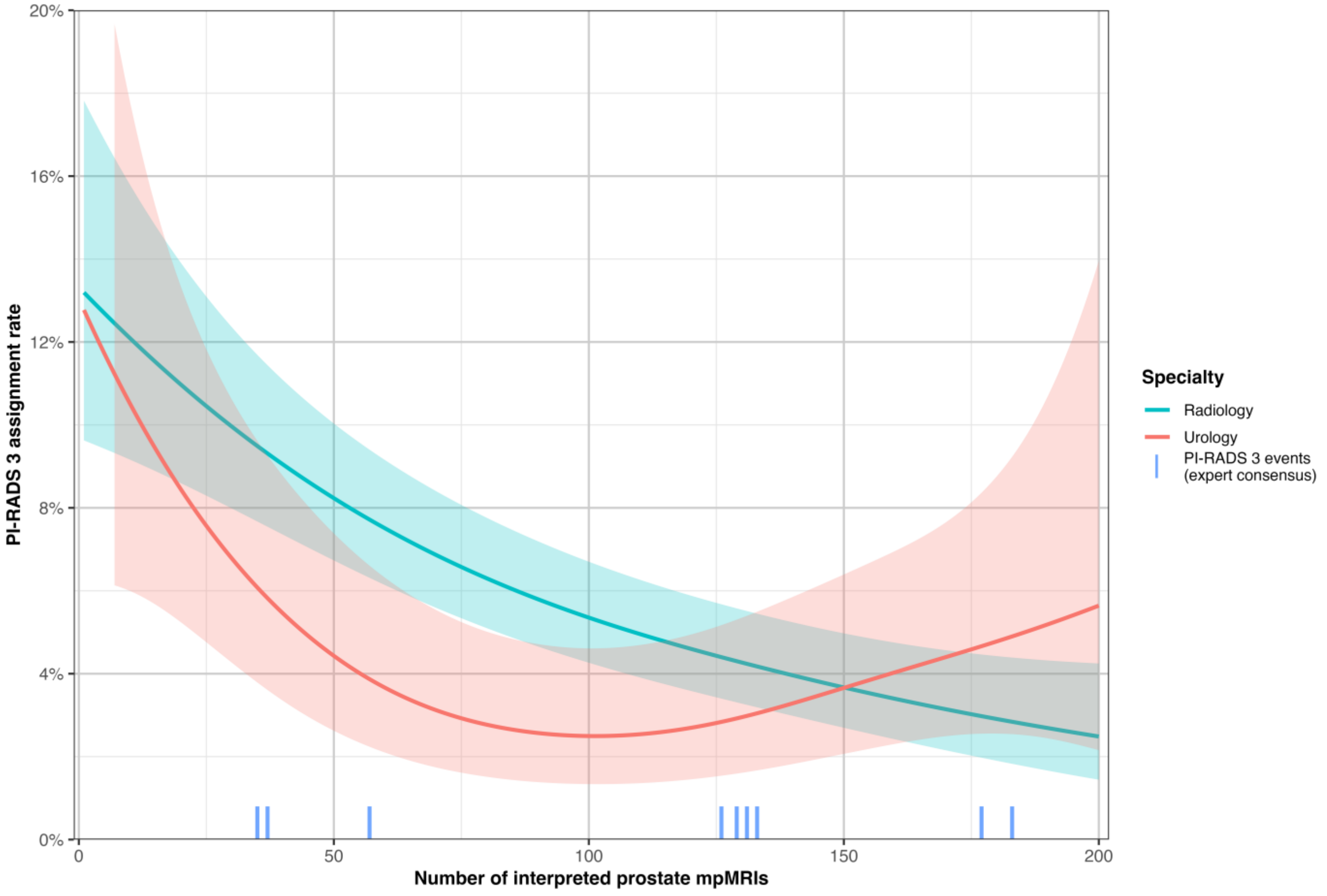

Supplement: Supplementary file 1 — ELECTRONIC SUPPLEMENTARY MATERIAL [file 330_2025_12177_MOESM1_ESM.pdf]
